# Kerion, an often missed scalp lesion: A case report

**DOI:** 10.51866/cr.719

**Published:** 2026-08-18

**Authors:** Sulaiman Suriani, Abdul Rahim Nazatul Shima

**Affiliations:** 1 Klinik Kesihatan Putrajaya, Presint 9, 1, Jalan P9e, Presint 9, Putrajaya, Wilayah Persekutuan Putrajaya, Malaysia.; 2 Putrajaya Hospital, Jalan P9, Presint 7, Putrajaya, Wilayah Persekutuan Putrajaya, Malaysia.

**Keywords:** Kerion, Tinea capitis, *Trichophyton*, Dermatomycoses

## Abstract

Kerion is an abscess caused by a fungal infection, commonly occurring on the scalp among children and rarely in adults. This report illustrates the case of a 12-year-old boy who presented with multiple plaques with crusty lesions on the scalp for 2 months, associated with purulent discharge and boggy swelling, initially misdiagnosed as seborrheic dermatitis. Following unsuccessful treatment, his condition was correctly diagnosed as kerion based on clinical appearance, history of contact with domestic animals and Wood’s lamp examination findings. The diagnosis was delayed for 2 months, leading to a prolonged disease course. Complete resolution of symptoms and lesions was achieved with systemic antifungal treatment, although permanent scarring alopecia remained. The delay in diagnosis and treatment led to prolonged illness and alopecia.

## Introduction

Tinea capitis is a common dermatophyte infection of the scalp that predominantly affects children, with causative organisms varying according to geographic region.^1,2^ It is mainly caused by *Trichophyton tonsurans* (endothrix) as well as *Microsporum canis* and *Microsporum audouinii* (ectothrix).^2,3^

Endothrix infections, such as those caused by *T. tonsurans,* invade the hair shaft while preserving the cuticle, resulting in hair breakage at the scalp surface and producing the characteristic ‘black dot’ appearance. Other examples of endothrix species include *Trichophyton rubrum, Trichophyton soudanense* and *Trichophyton violaceum.* In contrast, ectothrix infections, such as those caused by *M. canis,* form spores on the outside of the hair shaft, leading to destruction of the cuticle and, in some cases, fluorescence under Wood’s lamp.^3^

Kerion represents a severe inflammatory variant of tinea capitis, characterised by a boggy, tender plaque with associated hair loss and potential progression to permanent scarring alopecia if not treated promptly.^4,5^ In Southeast Asia, including Malaysia, zoophilic dermatophytes such as *M. canis* are commonly implicated due to frequent contact with domestic and stray animals and are associated with more inflammatory presentations such as kerion.^1^ This contrasts with many developed urban settings, where anthropophilic species such as *T. tonsurans predominate^1,6^*

This report highlights the case of a 12-year-old boy with kerion that was initially misdiagnosed as seborrheic dermatitis and bacterial infection, resulting in delayed diagnosis and treatment and ultimately leading to permanent scarring alopecia.

## Case presentation

A 12-year-old Malay boy presented with a 2-month history of scalp swelling, initially manifesting as a small, tender plaque on the occipital region that progressively increased in number and size, accompanied by purulent discharge. He had no previous medical issues and exhibited no constitutional symptoms such as fever, weight loss or appetite loss. The boy had been in contact with stray cats at hisgrandparents’ home, although it was unclear if thecats were healthy or had similar lesioas. No family members exhibited similar symptoms.

Over the 2-month period, he sought care at multiple healthcare facilities and was initially misdiagnosed with seborrheic dermatitis and later with bacterial infection, resulting in a significant delay in appropriate treatment. He was nreated with topical antieunoals and subsequently oral aloxacillin 500 mg QID without improvement and was than referred to a dermatology clinic.

**Figure 1:**
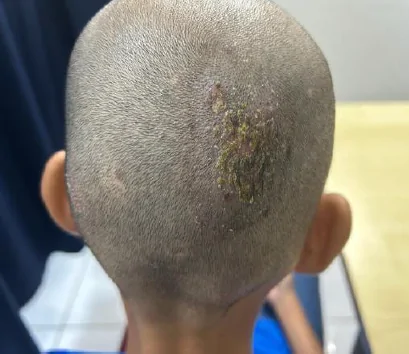
Boggy plaques with yellowish crusts on the parieto-occipital region.

**Figure 2:**
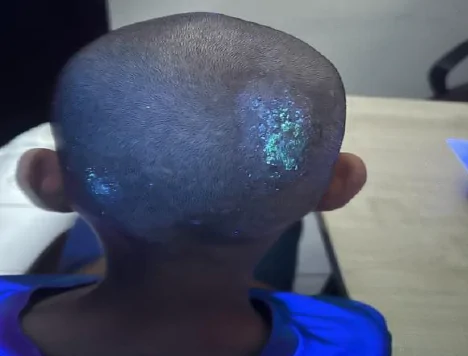
Wood’s lamp examination showad yeSow-green fluorescence over the affected region.

In the dermatology clinic,examination revealed ihree bogay plaques onthe parietal and occipital areas (as shown in Figure 1), the largest bein g a 5x4-cm crusted lesion on the right occipital area. Occipital and cervical lymph nodes were palpable, and there was notable hair loss over the affected areas. Wood’s lamp examination showed yellow-green fluorescence (as shown in Figure 2), indicative of ectothrix infection.^3^ Plucked hair samples were teat foa Ringaleulture.

Clinically a diagnosis of tinea capitis with kerion was made. Although initially labelled as having ‘secondary bacterial infection’, this was reconsidered as kerion itself, which can present with pus and bogginess without bacterial infection.^7^ Treatment included oral griseofulvin 375 mg OD (10-20 mg/kg/day) and biweekly ketoconazole 2% shampoo. He was advised to complete the course of oral cloxacillin 500 mg QID, which was initiated earlier. Topical miconazole cream was also added to the treatment regimen as an adjunctive treatment.^8,9^ Household contacts were examined, and no similar lesions were found.

Ater 2 weeks of treatment, there was marked clinied improvement, with reduction in inflammation and resolution of discharge. Following completion of a total of 8 weeks of systemic antifungal therapy the lesions resolved; however, residual scarring alopecia persisted over the affected areas, reflecting the consequence of delayed diagnosis.

## Discussion

Kerion is a severe inflammatory form of tinea capitis characterised by painful boggy scalp lesions, purulent discharge and regional lymphadenopathy. Early diagnosis and treatment are essential to prevent complications, including permanent alopecia, particularly in children.

Tinea capitis, primarily caused by the fungi T *tonsurans* and *M.* aantinvades keratinised tissues using keratinolytic enzymes, leading to keratin degraCation. Thisinfectaon predominantly affects children globally, with the highest incidence kbserved within 3–7 years of age.^2^ While it can appear in any age group, it is uncommon in adults unless they are immunocompromised. The prevalence and epidemiological characteristics of tinea capitis vary by region. For instance, a decade-long study in Iran revealed that tinea capitis was the most common dermatophyte infection, accounting for 52.7% of cases, followed by tinea corparis (24%) and tinea pedis (8.9%).^4^

Tinea capitis may not initially show symptoms b ut can develop into various forms as the infection progresses. The non-inflammatory type typicallo presents as fine scalins on distinct, scaly patcher of circular hair loss, resembling dandruff with subtle hair thinning, often accompanied by ‘black dots’ on the scalp from hair breakage.^2^

Severe inflammatory forms of tinea capitis include kerion and favus. Kerion presents as a tender, erythematous, pus-filled scalp swelling with alopecia and regional lymphadenopathy, reflecting a hypersensitivity reaction to the fungal infection.^5^ Although traditionally associated with zoophilic ectothrix infections, kerion is increasingly linked to endothrix infections such as *T. tonsurans,* particularly in urban settings.^6^

Tinea capitis is frequently misdiagnosed as a bacterial infection, resulting in unnecessary antibiotic or surgical treatment and delayed antifungal therapy, which may lead to permanent scarring alopecia. It is commonly mistaken for seborrhoeic dermatitis, atopic dermatitis, psoriasis, alopecia areata, furunculosis and trichotillomania.^7^ Misdiagnosis is often related to low clinical suspicion, limited awareness and inadequate diagnostic resources.^7-8^

Diagnosis is aided by Wood’s lamp examination and potassium hydroxide (KOH) microscopy. Ectothrix infections, such as *M. canis,* typically demonstrate bright green to yellow-green fluorescence, whereas endothrix infections, including T *tonsurans,* do not fluoresce. In primary care settings where these tools are unavailable, kerion may be diagnosed clinically based on boggy inflammatory scalp swelling, patchy alopecia, purulent discharge, cervical lymphadenopathy and animal exposure.^2,7^ Differential diagnoses include seborrhoeic dermatitis, bacterial infections, alopecia areata and psoriasis. Unlike kerion, seborrhoeic dermatitis presents with non-inflammatory greasy scales without significant hair loss, while alopecia areata causes well-demarcated, noninflammatory patches of alopecia.^2,7^ Psoriasis is distinguished by well-demarcated plaques with silvery scales.^2^ In this case, marked inflammation, tenderness and progressive alopecia were key clues supporting a diagnosis of kerion over seborrhoeic dermatitis.

A definitive diagnosis is established by culturing the fungus from hair and scalp samples. However, obtaining fungal samples from kerions may be difficult and frequently yields negative results due to intense inflammation.^9^ Thus, clinical judgement plays a pivotal role in the diagnosis and treatment of kerion.^9^ Additionally, dermoscopy serves as a sensitive diagnostic tool, capable of identifying specific hair shapes indicative of different fungi, including comma-shaped hairs for *T. tonsurans* and dystrophic, elbow-shaped hairs for *M. canis?*

In this case, the diagnosis of kerion was supported through clinical observation and the detection of yellow-green fluorescence under Wood’s lamp. The initial misdiagnosis as seborrheic dermatitis and folliculitis delayed the appropriate antifungal treatment because antibiotics were prescribed instead. The lack of supportive tools such as Wood’s lamp and KOH examination in primary healthcare settings further contributed to this misdiagnosis.

Systemic griseofulvin remains the primary effective oral treatment for tinea capitis, typically prescribed for a duration of 4–6 weeks. Topical treatments are generally ineffective when used alone for this condition.^9,10^ Other effective oral treatments include itraconazole, terbinafine and fluconazole.

Systemic antifungal therapy is essential for the treatment of tinea capitis and its inflammatory variant, kerion. Both griseofulvin and terbinafine are effective oral agents, with their efficacy influenced by the causative organism. Terbinafine has been shown to be more effective against *Trichophyton* species and is often preferred due to its shorter treatment duration, while griseofulvin remains more effective for *M. canis* infections.^11,12,13^ In paediatric patients, pharmacokinetics and safety profiles are important considerations. Griseofulvin has a long-established safety record but requires prolonged therapy and is better absorbed when taken with fatty meals, whereas terbinafine is fungicidal, accumulates in keratinised tissues and allows for shorter, once-daily dosing.^12^ However, prolonged use of griseofulvin may be associated with adverse effects such as gastrointestinal disturbances, headache, photosensitivity and, rarely hepatotoxicity or haematological abnormalities, necessitating monitoring during extended treatment.^11,12^ Itraconazole is another effective alternative and is available as an oral solution (syrup) with superior bioavailability compared with capsules, making it suitable for paediatric use; the recommended dose is approximately 3–5 mg/kg/day.^6,11^ Nevertheless, azole antifungals require caution due to potential drug interactions and hepatotoxicity.^12^

Adjunctive topical antifungal creams may provide symptomatic relief but should not be used as sole therapy, as dermatophyte infection of the hair shaft and follicles requires systemic antifungal treatment.^9,11,14^ Selenium sulphide or ketoconazole shampoo may be used 2–3 times weekly for 2–4 weeks to reduce spore shedding and transmission.^9-11^

Preventive measures are important to limit transmission among household contacts and schoolmates. Children receiving treatment may continue attending school but should avoid sharing personal items such as combs, hairbrushes and hats. Household contacts should undergo scalp examination and may use prophylactic antifungal shampoo for 2-4 weeks. Pets suspected of being infection sources should also be examined and treated appropriately.^10^

The prognosis of tinea capitis is generally favourable, with most patients achieving complete recovery and hair regrowth. However, severe inflammatory forms such as kerion and favus may result in permanent scarring alopecia if treatment is delayed. Regular follow-up is recommended to ensure clinical resolution, monitor hair regrowth and reduce the risk of recurrence.^10,11^

## Conclusion

In conclusion, kerion is frequently misdiagnosed as a bacterial infection because of its inflammatory presentation. Clinicians should consider fungal infection in children with inflammatory scalp lesions, particularly when associated with hair loss, animal exposure, or poor response to antibiotics. In resource-limited settings, empirical systemic antifungal therapy may be justified based on clinical suspicion and treatment response. Early recognition and treatment are essential to prevent permanent scarring alopecia and other long-term consequences.

This case was limited by the absence of fungal culture confirmation, with the diagnosis based primarily on clinical findings and Wood’s lamp examination. It also highlights the diagnostic challenges faced in primary care when access to mycological investigations is limited. Maintaining a high index of suspicion and initiating timely antifungal therapy or referral can significantly reduce morbidity and prevent complications.
